# Twitch Data in Health Promotion Research: Protocol for a Case Study Exploring COVID-19 Vaccination Views Among Young People

**DOI:** 10.2196/48641

**Published:** 2023-10-18

**Authors:** Brian Chau, Melody Taba, Rachael Dodd, Kirsten McCaffery, Carissa Bonner

**Affiliations:** 1 Sydney Health Literacy Lab School of Public Health, Faculty of Medicine and Health University of Sydney Sydney Australia

**Keywords:** twitch, social media, COVID-19, vaccination communication, video gaming, gaming, health promotion, streaming

## Abstract

**Background:**

Social media platforms have emerged as a useful channel for health promotion communication, offering different channels to reach targeted populations. For example, social media has recently been used to disseminate information about COVID-19 vaccination across various demographics. Traditional modes of health communication such as television, health events, and newsletters may not reach all groups within a community. Health communications for younger generations are increasingly disseminated through social media to reflect key information sources. This paper explores a social media gaming platform as an alternative way to reach young people in health promotion research.

**Objective:**

This protocol study aimed to pilot-test the potential of Twitch, a live streaming platform initially designed for video gaming, to conduct health promotion research with young people. We used COVID-19 vaccination as a topical case study that was recommended by Australian health authorities at the time of the research.

**Methods:**

The research team worked with a Twitch Account Manager to design and test a case study within the guidelines and ethics protocols required by Twitch, identify suitable streamers to approach and establish a protocol for conducting research on the platform. This involved conducting a poll to initiate discussion about COVID-19 vaccination, monitoring the chat in 3 live Twitch sessions with 2 streamers to pilot the protocol, and briefly analyze Twitch chat logs to observe the range of response types that may be acquired from this methodology.

**Results:**

The Twitch streams provided logs and videos on demand that were derived from the live session. These included demographics of viewers, chat logs, and polling results. The results of the poll showed a range of engagement in health promotion for the case study topic: the majority of participants had received their vaccination by the time of the poll; however, there was still a proportion that had not received their vaccination yet or had decided to not be vaccinated. Analysis of the Twitch chat logs demonstrated a range of both positive and negative themes regarding health promotion for the case study topic. This included irrelevant comments, misinformation (compared to health authority information at the time of this study), comedic and conspiracy responses, as well as vaccine status, provaccine comments, and vaccine-hesitant comments.

**Conclusions:**

This study developed and tested a protocol for using Twitch data for health promotion research with young people. With live polling, open text discussion between participants and immediate responses to questions, Twitch can be used to collect both quantitative and qualitative research data from demographics that use social media. The platform also presents some challenges when engaging with independent streamers and sensitive health topics. This study provides an initial protocol for future researchers to use and build on.

**International Registered Report Identifier (IRRID):**

RR1-10.2196/48641

## Introduction

### Role of Social Media in Health Promotion

Social media platforms have emerged as influential channels for health communication, facilitating health promotion and information dissemination to social media users. Social media has been effectively used across varied health promotions’ areas including health care decision-making and to promote healthy behaviors and interprofessional communication [1-3]. Social media’s impact has been the result of several factors including its broad reach, active engagement by users, and support of multimedia content [1,2]. Social media provides an opportunity to support health promotion, with effective data dissemination enabling more cost-effective and wider reaching health campaigns [1]. It also enables the development of live-streamed content and graphics related to health promotion, which may be more engaging for certain populations [2].

For example, Laranjo et al [4] investigated the use of social media in promoting physical activity. The paper illustrated how social media platforms can engage a wide community and facilitate the interactions with users to motivate healthier choices and behavior change. Health interventions on platforms such as Facebook and Twitter were found to have a positive effect on health behavior outcomes. Similarly, Moorhead et al [5] highlights the role of social media in health information dissemination through examining the potential for collaboration between users and health professionals. It provided rapid communication avenues to aid health authorities in a timely and targeted manner, however it requires careful monitoring and guidance to ensure quality, reliability, and privacy [5]. Timely and effective communication is a significant concern during health crises, such as the COVID-19 pandemic. The pandemic illustrated the usefulness of these platforms for rapid dissemination of information, but also the pitfalls of misinformation [6]. Misinformation and antivaccination sentiments have been widespread and infrequently moderated on social media platforms [7], despite the growing rate of people acquiring their news through platforms such as Facebook and Twitter [8,9].

Further, 1 group that may particularly benefit from health promotion via social media are younger people. With an increasing proportion of the younger population accessing and using social media [10,11], modes of health communication need to adapt in order to actively engage with the community. Without social media engagement, health communications may not reach all members of the community.

### Potential of Twitch as a Health Promotion Platform

Twitch has been recognized as the most successful and popular web-based streaming platform across the globe [12]. With over 200 million viewers, social media influencers have amassed large audiences for content spanning from gaming to comedy [12]. Although social media has been used by the government in health communications [13,14], Twitch has potential as an effective additional platform to reach young people due to the target demographic of many of the games and activities that are streamed. The 2-way interactions between streamer and viewer on this platform present a new opportunity for research and health promotion.

Woodcock and Johnson [12] conducted a recent analysis of viewers’ experiences of parasocial interactions with videogame streamers on Twitch. The research highlights a male-dominated but mixed audience across the platform and how streamers that actively interact with their viewers in a context separate to health are less likely to engage in inflammatory, irrelevant, or disruptive comments [12]. Harpstead et al [15] highlighted the need for understanding the approaches used in social media research and produced a framework for a research toolkit for streaming platforms through a systematic review. This study outlines the primary methods of data acquisition on Twitch such as log data analyses and survey methods and the data types that can be used [15]. They also found a lack of research that studies the relationship between streamers and their audiences [15].

### COVID-19 Vaccination as a Topical Case Study

The COVID-19 pandemic has had a huge impact across the world over the last 3 years. While early international border closures and movement restrictions protected Australia at the start of the pandemic before vaccines were available, there were still many deaths after the spread could not be controlled [16]. At a time of low vaccination rates, the 2021 Delta variant started the first large uncontrolled outbreak in Australia, leading to further border closures and lockdowns [17]. Late 2021 and early 2022 saw the COVID-19 response in Australia largely shift toward vaccination as the primary prevention response to the Omicron subvariants [18]. The majority of Australians received their initial vaccination and a second dose [16]. However, booster vaccination dosage rates were much lower [16,17]. Misinformation continued to linger in the web-based community [19,20] and the needs of people with lower health literacy were inadequately met, leaving much to be improved in health communications.[20,21] At the time of this study, this was the most topical case study to use to explore the potential of Twitch for health promotion research.

### Aims

Twitch has been under-used for health promotion and research, but presents an opportunity to engage young people and gather unique perspectives on health issues. This protocol study aimed to pilot-test the potential of using Twitch to conduct health communication research with young men, using COVID-19 vaccination as a topical issue at the time of this study.

## Methods

### Preliminary Discussion and Protocol Development

In preparation to run a session with a streamer, the research team conducted a series of discussions with a Twitch Account Manager to confirm the requirements from the research team and Twitch. Through this iterative process, we established a preliminary framework for a protocol that could be used for gathering quantitative and qualitative data from Twitch. This included a strategy to identify an appropriate streamer to run a real time live video session about COVID-19 vaccination, a Twitch protocol for the streamers to run the session and coordination of the retrieval of Twitch data logs. This informed the development of a participant information sheet, consent form, and ethics application to proceed with the live sessions.

### Ethical Considerations

Ethical approval was provided by the University of Sydney Human Research Ethics Committee (#2021/880), based on identified Twitch Account Managers who received reimbursement for their time (AUD $100 [US $64.23 on September 21, 2023] voucher), and anonymous stream participants. Managers gave written consent after discussion about this study and reading information about this study. Stream participants were informed of this study at the start of the stream and a representative of the research team was available to answer any questions in the chat. Consent was indicated by participation in the poll and chat. The ethics committee raised some concerns about the potential for children to be identified through public Twitch records, but agreed to approve the protocol if no minors were identified in published quotes through usernames (eg, if birth year was included), and a link to reliable COVID-19 vaccination information was required to be posted at the end of the chat. The link was to the official government information about COVID-19 at the time of this study.

### Data Analysis

Once the live stream had been conducted and the Twitch Logs had been retrieved, the chat data was examined to describe the range of comment types provided in response to the poll on COVID-19 vaccination. The Twitch chat log data was pulled by the Twitch Account Manager, which is also available publicly, and sent through to the research team for thematic coding. These data were compiled as an excel sheet with timestamped comments from the stream. It should be noted that these descriptive categories were for data illustration purposes only and detailed thematic analysis was not the aim of this protocol paper. It intends to show the different data types that can be drawn from Twitch for health promotion research. As this project was focused on developing a methodological approach rather than a detailed qualitative analysis, an in-depth analysis was beyond the scope of what was required. However deeper conversation-based analysis could be conducted in future studies.

## Results

### Overview

In total, 1 session was conducted as a pilot protocol to identify what data types were possible to extract from the stream, followed by a 2 sessions run with a separate streamer to further test the refined protocol. The data gathered from the session can be separated into 3 components: results from the in-stream polls, live chat data from the Twitch logs, and viewer numbers and demographics provided to the streamer. Each is derived through a different mechanism and was discussed with the Twitch Account Manager prior to the live session to coordinate its retrieval.

### Participants

Table 1 provides an overview of the sessions that were conducted on Twitch and the usage of the platform.

**Table 1:** Number of views and chat log responses.

Livestream	Number of viewers (average across vaccine section of stream)	Number of poll responses	Number of chat log responses (relevant responses during vaccine section of stream)
Pilot	11	8	N/A^a^
Livestream 1	23	17	45
Livestream 2	15	9	2

^a^N/A: not available.

### Pilot Response

The pilot session was conducted to test the feasibility of the protocol for the live sessions and only included the polling component of the project. This was well received by the pilot session’s audience with over 70% (n=34) of viewers across the 3 livestreams responding to the poll (total viewers, n=49). As the stream was primarily a small gaming stream, the number of attendees was small with minimal live conversation, however this step was useful in scoping the visual presentation of a live stream and provided a demonstration of what tools were available. This led to a modified protocol for the live sessions, including prompting the poll to the audience, discussing potential chat starters, and access to stream resources such as demographic data and recording.

### Poll Data

**Figure 1:**
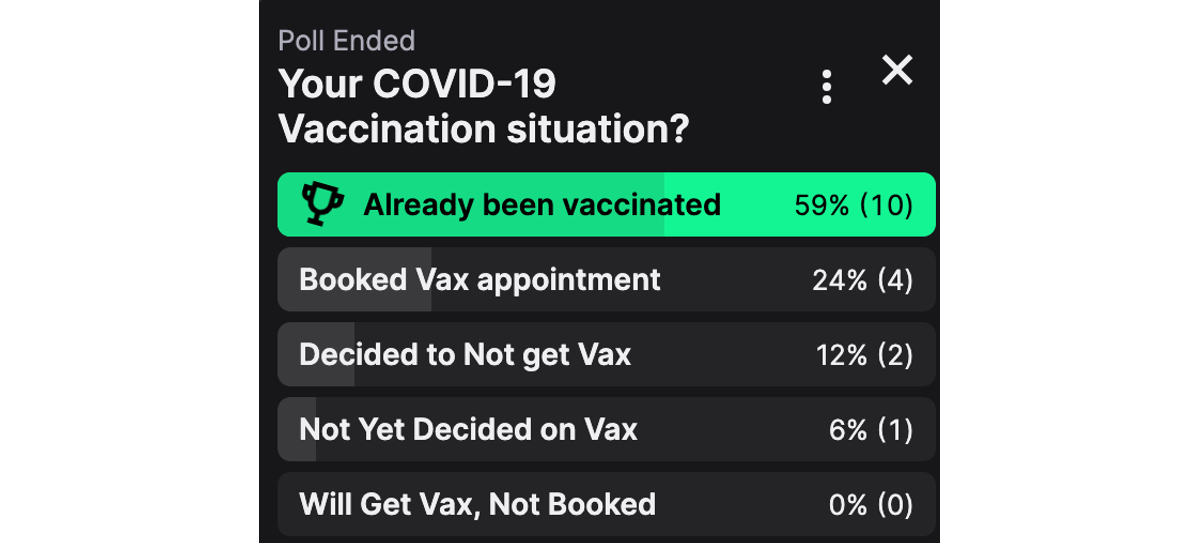
Example live session poll results.

**Figure 2:**
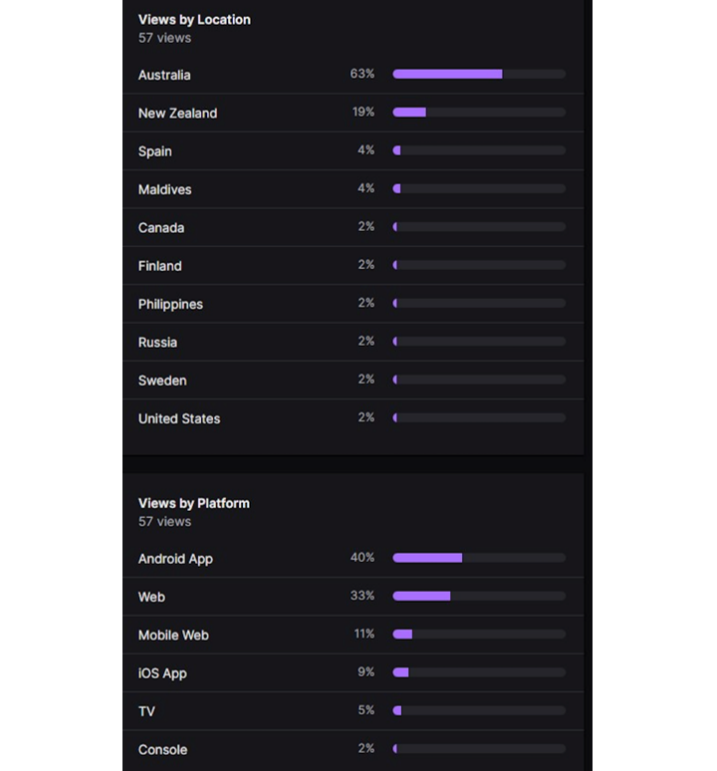
Demographics screenshot from Twitch Account Manager.

Each live session commenced with a brief introduction from the streamer followed by a poll that was prepared by the Twitch Account Manager to establish the vaccine hesitancy conversation (see Figure 1). The pilot and first session were conducted in August and September 2021, when booster shots had not been fully introduced, resulting in a set of poll questions focused on current vaccination status and intent to vaccinate. These questions ranged from “decided not to get vax” to “already been vaccinated,” as seen in Figures 1 and 2. The wording of the poll was discussed with the Twitch Account Manager, where the use of “vax” was interpreted to be easier to incorporate onto the Twitch platform. This set of questions continued over to the first live session and yielded provaccine sentiments (either vaccinated or have already booked vaccination appointment), reflecting higher numbers of individuals who had received the vaccination. The new set of poll questions revolved around booster and unvaccinated status to correlate with the shift in COVID-19 context in Australia. Most responders answered with a vaccinated and boosted status.

### Chat Log Data

The primary data yielded from both the pilot and live sessions was the Twitch chat log data, which is a recording of each message that is sent to the public livestream (see Textbox 1). This was composed of extracted spreadsheets of responses from Twitch viewers that attended each live session in response to the streamer’s questions and comments during a discussion phase. Content of each stream has been limited by setting time parameters on the retrieved spreadsheet. This is a necessary step to filter out content delivered by the streamer that is not relevant to the intervention. Twitch chat logs were not retrieved from the pilot session as the qualitative approach had not yet been developed. However, chat logs from both live sessions were retrieved through the Twitch Account Manager. It was derived from the Twitch Account Manager that this is possible without the support of the Twitch Account Manager; however, this study did not require this. Responses were initiated through the streamer openly discussing their own vaccination status, perspectives, and freely responding to messages delivered in the chat. This created an open-platform discussion and yielded unfiltered perspectives around the vaccine. Overall, Twitch chat logs revealed a range of both positive and negative responses and varying engagement in the health promotion topic. In this case study, response categories included irrelevant comments, false information, comedic and conspiracy responses, vaccine status, provaccine comments, and vaccine hesitant comments. See Table 2 for an example of a chat log extract and the varying categories these quotes fit into.

**Live Twitch session protocol.**

- Identify streamer that has interest in participating in this study.
- Participant information sheet to be sent through to the streamer.
- Conduct debrief session with streamer to discuss potential conversations and direction of Twitch live session to cover any issues or concerns from the participant information sheet.
- Researcher to join stream at a coordinated time under Sydney Health Literacy Lab Twitch account. At the start of stream, researcher to send through: “The poll and chat in this stream will be publicly available as per Twitch policy and will be used for research purposes as part of the Sydney Health Literacy Lab COVID-19 project. A summary of the results will be provided here: https://sydneyhealthliteracylab.org.au/projects/.”
- Throughout the stream, researcher to monitor the chat but should not intervene so as to influence the direction of the conversation during the livestream.
- At the end of the stream, researcher to send through: “If you have symptoms or questions about COVID-19, call the National Coronavirus Health Information Line on 1800 020 080. If you require translating or interpreting services, call 131 450. You can also find reliable information here: https://www.healthdirect.gov.au/coronavirus.”
- Twitch contact to send through an Excel (Microsoft Corp) spreadsheet of Twitch chat log for analysis.

**Table 2:** Example Twitch chat log and themes from a 1-minute excerpt first live session.

Theme	Chat log quote
Comedic	His 5g is great now
Provaccine	Novavax coming out in november I think
Vaccine status	Me. Im in wa. I know how science and statistics work.
Irrelevant comments	They and I
Comedic	Can confirm no 5G I am sad
Irrelevant comments	Want to know Y
Vaccine status	Me im not allowed ot get vax yet
Irrelevant comments	It looks like a highlighter
Conspiracy	2009 fine by the FDA. They paid 2.3 BILLION us.

### Live Twitch Session Protocol

During the live streams, the streamer replied to polling results as they were submitted. However, responses such as *“*am vaxxed and boosted. No spicy cough yet*”* were provided without direct questioning from the streamer, illustrating that the polling visual was sufficient to generate a response. It should be noted that there were some viewers who had difficulty in finding where the poll was, evident in *“*poll is not on [their] screen.*”* However, Twitch viewers can read and respond to other viewers responses and have the capacity to directly refer to another viewer’s response. Furthermore, streamers often read the chat and responded promptly to questions posted in the chat. The streamer for the live sessions outlined where the poll could be found shortly after it was asked and facilitated further discussions where possible. This was further supported by other members in the audience, with some answering *“*@[username] Poll is at the top of chat.*”* This functionality extended throughout the stream, with viewers frequently replying to each other’s responses and questions in a conversational tone, which can be seen throughout the Twitch chat logs. This produced a comprehensive collection of responses and perspectives on COVID-19 vaccines that would otherwise be difficult to retrieve. However, audio discussion was not recorded.

The Twitch chat logs demonstrated a range of responses to the health promotion topic even with the small case study sample. Many responses were irrelevant and directed primarily at other components of the stream, such as “sweet dreams are made of you” and “it looks like a highlighter.” These comments are either prompted by the streamer or another viewer. False information (ie, not consistent with official health authority information at the time), comedic and conspiracy responses were also prevalent, including responses such as “I’m taking the vaccine out if my 5G doesn’t go up” and “[will get the vaccine when] Pfizer isnt the company that paid the single largest criminal fine.” Relevant responses were categorized into vaccine status, pro vaccine, and vaccine hesitant comments and were comprehendible. Examples of vaccine status responses include “been vaccinated for over a month now, Biontech for two months now” and “I chose to get vaxxed but [I don’t know why].” Vaccine hesitant comments included “I don’t want to get the vaccine because I know it hurts” and “I won’t get the vaccine unless I get covid.” As there is no filter and minimal chat restrictions, open responses from the audience may lead to difficulty in interpreting results as colloquial abbreviations and terms were used on Twitch such as “idk” or “pog.” The Twitch Account Manager was aware of such difficulties and alluded to being able to support in interpreting these responses. Further analysis into these responses could be done in conjunction with videos on demand (VODs) as they will provide the contextual background to the prompt or question being asked by the streamer.

### Viewer and Streamer Demographics

The Twitch Account Manager also provided the demographics of live session streams (see Figure 2), providing insights into viewer country and platform that the stream was accessed from, however, this did not provide information on other demographic information such as gender or age. This information was not provided by Twitch to the research team even though this information is required to create an account. Further information on viewer demographics could be gathered by consulting the streamer directly. This was not conducted for this study but could be considered in future studies to ascertain further viewer demographics. Figure 2 is a graphical representation of the demographic data that is automatically collected by the Twitch streaming platform after each stream. These data were collected through the Twitch Account Manager or streamer and is not available to the public. An additional facet that can be considered is the selected streamer. The content available on Twitch is separated into several categories. Provided the limited sample size and potential opportunities, a selection criterion for the streamer was not a major consideration for this study; however, an effort was made to expand potential streamers. Through this process, several different demographics were considered, such as female streamers, gaming streamers, comedians, and “in real life” streamers. This will be explored further in the discussion.

### VODs

Recordings of the sessions are saved to the Twitch platform and can be accessed through the streamer’s profile page if this function is enabled. For this project, a recording of the session was provided by the Twitch Account Manager through a link which expired shortly after it was received. Due to difficulties in contacting the Twitch Account Manager once they moved to a different role, this was not followed up on as it was not necessary for the purposes of this project. For future studies, this can be avoided by establishing clearer requirements from both Twitch and the research team.

## Discussion

### Summary of Findings

This study aimed to develop and test a protocol for using Twitch to engage with young people for health promotion research. The live sessions commenced with a poll to establish the conversation, which focused on COVID-19 vaccination as the most topical health promotion issue at the time of this study. The primary data collected were the Twitch chat log data, which demonstrated a range of responses to and engagement with the health promotion topic. The demographics of live session streams provided insights into viewer country and cross-platform usage of the Twitch viewership, which illustrates the reach of the session. Recordings of the sessions were saved on the Twitch platform and can be accessed through the streamer’s profile page if this function is enabled, however this was not used for the purposes of this study.

### Future Potential and Challenges of Using Twitch

Discussions about conducting research on the Twitch platform started with the Twitch Account Manager on Twitter, as there was limited prior research to inform the ethics application and protocol. Several issues needed to be considered, including addressing the fact that children older than 13 years can create an account and view streams. Additionally, there is no age verification procedure on Twitch, making it challenging to ensure minors are not participating. Procedures to minimize engagement with people under 18 through eligibility information can be integrated into this study’s design, but they are not foolproof. Ownership of Twitch chat log and VOD data was also an issue that needs to be considered by future researchers. Twitch owns all Twitch data for the first 48 hours after the livestream to promote engagement on the platform. After 48 hours, the data become publicly available and accessible at the streamer's discretion. Access to both the chat logs and VODs could be obtained by communicating with the streamer, but streamers can choose to enable or disable VODs. Therefore, it is important to discuss this with the streamer prior to the session.

Both qualitative and quantitative types of data can be extracted from Twitch live streams. The primary data source explored for this study was open ended discussions and conversations that were recorded in the Twitch chat logs. These data are useful for providing anonymous and unbiased responses from the target population, however more importantly, it provides a platform for health promoters to observe how individuals might interact and respond to others on the internet about a specific topic. This could be used to develop health promotion material using language appropriate to the target group, and can also provide an opportunity for referral to reliable information and health expert input when requested. For research purposes, a link to the research team’s website and official government information about the health topic were required for ethical approval. The uptake and impact of such referrals could be explored in future research.

Finally, open-ended responses from communities viewing content on Twitch may lead to inflammatory conversations that could harm the perspectives and emotions of other viewers. The guidelines for managing inflammatory comments on each social media platform varies and can change over time.

### Strengths and Limitations

This paper provides a feasible protocol for using a novel method in health promotion research, to inform future research in this area. Several improvements for future researchers were identified during the project. Limitations of the protocol included reliance on the availability of the Twitch Account Manager and streamer, which sometimes led to delays and limits on data access and ownership. When this presents significant barriers to researchers, using publicly available Twitch data from naturally occurring Twitch streams and chats could be considered for analysis. However, this would limit the ability to direct the conversation to a specific health promotion topic.

In future research, the selection of suitable Twitch Account Manager and streamers given this study’s aims should be given consideration and prioritized before conducting any live sessions. As this was a case study only, the sample size and diversity of streamers are limited. The Twitch platform provides a variety of contextual and demographic opportunities for future research. For example, responses from a comedy-focused male streamer may differ significantly from those of a gaming-focused female streamer.

Textbox 2 provides a summary of recommendations for future health promotion research using Twitch.

**Recommendations for health promotion research using Twitch.**

**Recommendations for future research on Twitch**

- Minimize communication issues by addressing methodological and ethical concerns (eg, how to deal with inflammatory comments in accordance with the platform policies) prior to engagement with Twitch. Provide resources, forms, and approvals early on to avoid delays.
- Establish communication strategies with the Twitch Account Manager and streamer using appropriate channels such as Discord or Twitter.
- Discuss all requirements, including consent, video on demand recordings, Twitch chat logs, and demographic data before running live sessions. These data should be permanently available and retrieved soon after the livestream.
- Engage a more diverse sample of Twitch Account Managers and streamers to improve the reliability and validity of data gathered.
- Analyze video on demand data in addition to Twitch chat log data to obtain a comprehensive data set including streamer input.

### Conclusions

The emergence of new social media platforms has led to the expansion of health promotion strategies beyond traditional methods. This study developed and tested a feasible protocol showing the potential of Twitch as a novel channel for health promotion research with young people. For research, the platform can be used to produce quantitative and qualitative data, but also presents some challenges when engaging with independent streamers and sensitive health topics. This study aims to offer guidance for future research and practice by providing a protocol for researchers to use and build on.

## Abbreviations

VOD: Video on demand
